# The Potential of Surveillance Data for Dengue Risk Mapping: An Evaluation of Different Approaches in Cuba

**DOI:** 10.3390/tropicalmed8040230

**Published:** 2023-04-18

**Authors:** Waldemar Baldoquín Rodríguez, Mayelin Mirabal, Patrick Van der Stuyft, Tania Gómez Padrón, Viviana Fonseca, Rosa María Castillo, Sonia Monteagudo Díaz, Jan M. Baetens, Bernard De Baets, Maria Eugenia Toledo Romaní, Veerle Vanlerberghe

**Affiliations:** 1 Epidemiology Department, “Pedro Kourí” Institute of Tropical Medicine, Havana 11400, Cuba; 2 Unidad de Información y Biblioteca, Instituto de Ciencias Nucleares, Universidad Nacional Autónoma de México, Ciudad de México 04510, Mexico; 3 Faculty of Medicine and Health Sciences, Ghent University, 9000 Ghent, Belgium; 4 Centro Provincial de Higiene Epidemiología y Microbiología, Dirección Provincial de Salud, Santiago de Cuba 90100, Cuba; 5 Unidad Provincial de Vigilancia y Lucha Antivectorial, Dirección Provincial de Salud, Santiago de Cuba 90100, Cuba; 6 Centro Provincial de Higiene Epidemiología y Microbiología, Dirección Provincial de Salud, Cienfuegos 55100, Cuba; 7 KERMIT, Department of Data Analysis and Mathematical Modelling, Ghent University, Coupure Links 653, 9000 Ghent, Belgium; 8 Public Health Department, Institute of Tropical Medicine, Nationalestraat 155, 2000 Antwerp, Belgium

**Keywords:** dengue, arbovirus, *Aedes*, epidemiology, prevention, stratification, risk mapping, Cuba

## Abstract

To better guide dengue prevention and control efforts, the use of routinely collected data to develop risk maps is proposed. For this purpose, dengue experts identified indicators representative of entomological, epidemiological and demographic risks, hereafter called components, by using surveillance data aggregated at the level of Consejos Populares (CPs) in two municipalities of Cuba (Santiago de Cuba and Cienfuegos) in the period of 2010–2015. Two vulnerability models (one with equally weighted components and one with data-derived weights using Principal Component Analysis), and three incidence-based risk models were built to construct risk maps. The correlation between the two vulnerability models was high (tau > 0.89). The single-component and multicomponent incidence-based models were also highly correlated (tau ≥ 0.9). However, the agreement between the vulnerability- and the incidence-based risk maps was below 0.6 in the setting with a prolonged history of dengue transmission. This may suggest that an incidence-based approach does not fully reflect the complexity of vulnerability for future transmission. The small difference between single- and multicomponent incidence maps indicates that in a setting with a narrow availability of data, simpler models can be used. Nevertheless, the generalized linear mixed multicomponent model provides information of covariate-adjusted and spatially smoothed relative risks of disease transmission, which can be important for the prospective evaluation of an intervention strategy. In conclusion, caution is needed when interpreting risk maps, as the results vary depending on the importance given to the components involved in disease transmission. The multicomponent vulnerability mapping needs to be prospectively validated based on an intervention trial targeting high-risk areas.

## 1. Introduction

Incidence of diseases, such as dengue, Zika and chikungunya, which are significantly contributing to the global burden of diseases, are steadily expanding in the world [1]. In particular, dengue is caused by one of four dengue virus serotypes (DENV-1, DENV-2, DENV-3 and DENV-4) belonging to the *Flaviviridae* family [2]. The control of arboviruses and also other viral diseases with epidemic potential becomes progressively more difficult, and struggles with increasing costs of control interventions and, in the case of vector control programs, increasing insecticide resistance [3]. It has been repeatedly highlighted that there is a critical need to develop novel approaches to prevent and/or control these diseases [4,5,6,7].

As transmission of infectious diseases, specifically vector-borne ones, is highly heterogeneous in space and time [8], several researchers as well as the World Health Organization [9] proposed a better use of routinely collected surveillance data, for instance through a risk mapping tool, to guide timely and effective management of outbreaks [10,11]. This is possible in Low and Middle Income Countries (LMIC), where disease incidence, disease-related risk factors and surveillance data are often available on a weekly or monthly basis, mainly from public health services. The system most often used for systematic reporting with a nation-wide coverage is the District Health Information System version 2 (DHIS2) [12].

Spatial and temporal risk mapping based on surveillance data is not only important for arboviral disease prevention but also for other infectious diseases, such as malaria and COVID-19, where control measures are intensified in a geographical area when an epidemic occurs, measured through test-positivity rate, case counts or hospitalization rates exceeding a threshold [13,14,15]. Additionally, for arbovirus control, risk mapping allows for guiding preventive measures complementary to the widely applied control efforts reactive to an outbreak. Indeed, current vector control strategies in dengue-endemic countries—*Aedes* foci detection, environmental management, larvicide application and adulticide spraying—are mainly carried out/intensified in response to detected clinical cases but fail to contain outbreaks or the spread of emerging *Aedes*-borne diseases such as chikungunya or Zika. Such a reactive outbreak response means in practice that actions are implemented several days or weeks after infection occurred—usually near or after the peak of an epidemic [16]—and are directed towards the case households, which may not be the major sites of infection given the high daytime mobility of the population [17,18].

An enhanced approach, supported by theoretical evidence, focuses on the identification of areas that concentrate a large fraction of *Aedes*-borne disease cases or elevated transmission risk for reframing vector control actions [19]. This will allow control programs to target their interventions to the areas at highest risk of transmission and, hence, increase the quality while decreasing the cost of the interventions and also decrease the use of insecticides, which in itself affects insecticide-resistance development of the involved vectors [18].

Risk mapping for arboviruses exists in the shape of single-component temporal or spatial models using clinical case reports [20,21,22,23]. The traditional approach to measure a relative disease risk uses the Standardized Incidence Ratio (SIR). However, this approach has been found to be instable, especially in sparsely populated areas [24]. Alternative solutions have been proposed to overcome this lack of reliability and also to take into consideration the effect of other risk (and/or protective) factors [25].

Clinical dengue case incidence is a proxy for transmission risk [26], however, there are many asymptomatic cases, which are responsible for silent and cryptic transmission of arboviruses. In order to take this aspect into account, the accuracy of risk models can be improved by including, besides case data, other factors influencing transmission, such as population density, entomological infestation and environmental and social characteristics [17,27,28,29,30,31]. Such multicomponent approaches for dengue risk mapping can be based on indices or on models [27]. For the former, vulnerability indices have been proposed based on exposure and susceptibility indicators [32], which typically integrate multiple indicators in a single measure. They generally rely on the aggregation of data of different indicators using a weighted summation.

Several vulnerability frameworks have been developed and later adapted for health risk assessment. For example, the European research project MOVE (Methods for the Improvement of Vulnerability Assessment in Europe) [33] created a framework for the study of climate change that has been adapted to assess socioeconomic vulnerability to dengue fever in Cali, Colombia [30]. In Brazil, the Health Vulnerability Index (HVI) developed by Gerencia de Epidemiologia Informaçao (GEEPI, 2013) Belo Horizonte and the ArboAlvo model have been used for the study of dengue and other arboviral diseases [34,35]. At a global scale, maps of vulnerability to infectious diseases have been created using methodologies such as the Infectious Disease Vulnerability Index (IDVI) [36] and the Water-Associated Disease Index (WADI) [32]. The WADI has been used for dengue vulnerability estimation and mapping at different spatial scales in several countries [32,37,38,39,40]. This index has been widely used because of its ease of implementing and because it is constructed using freely available data such as living conditions, population characteristics, climate, land use and land cover [32].

The knowledge-based multicomponent index that we present here, different from the above approaches as it only using routine data at the local level, takes into account several indicators describing the multiple dimensions of the complex web of factors influencing the transmission of dengue and affecting the susceptibility of the population and the exposure to the vector transmitting the virus (the agent). The components and indicators included were proposed by experts and technical staff active in vector control programs and in epidemiological research. The index is not only built based upon their experience but also takes into account the evidence of the published literature and the availability of the data.

Alternatively, generalized linear mixed modeling is an established tool to obtain disease risk estimates and maps, which may improve local estimates by incorporating spatial correlation and the effects of explanatory variables [41]. The variety of the results given by several approaches may not necessarily be a symptom of wrongful implementation, but of different views of a complex problem. A balance should be sought to design the simplest, yet most accurate, model that can be used in a routine setting to guide disease control efforts. A compromise must be found between the level of empirical detail needed, the availability of information and the potential applicability for the design of vector control interventions [42].

In this study, we aim to describe methods to identify areas of higher vulnerability for dengue transmission. This can be used by control programs to guide prevention and control strategies targeting high-risk areas. Therefore, we present different approaches, incidence- and vulnerability-based, of single- and multicomponent risk mapping based on surveillance data that are available at a decentralized level (the level of decision making for control actions) in Cuba.

## 2. Materials and Methods

### 2.1. Design

We design a methodological study to compare multicomponent risk models with single-component models for disease occurrence and evaluate the added value of the former. We illustrate the application of this methodology through a case study in two Cuban settings.

### 2.2. Settings and Data Collection

Settings. For this case study, we selected Cienfuegos and Santiago de Cuba, two cities in Cuba with a different history of dengue transmission and context. These two municipalities are the capital cities of their respective provinces. The spatial unit of analysis was the Consejo Popular—hereafter called CP, in plural, CPs—which is a local administrative structure with representatives of the government, community organizations and the health sector, among others [43].

The Santiago de Cuba municipality, located in the southeast of Cuba, has a population of 506,037 inhabitants and a population density of 490.5 inhabitants/km^2^ [44]. There are 19 urban CPs, with an average population of 15,035 inhabitants. The climate is warm with high temperatures (28–34 °C) and little rainfall, mainly between June and September. Between 1997 and 2010, the four serotypes of dengue caused large but controlled outbreaks, namely in 2001 (less than 50 cases), 2006–2007 (approximately 13,000 cases) and 2010 (approximately 2600 cases). Since 2010, the transmission pattern is mostly endemo-epidemic and cases are reported every year [45,46,47,48].

The Cienfuegos municipality, located in the center-southern part of Cuba, has 171,946 inhabitants and a population density of 483.5 inhabitants/km^2^ [44]. There are 19 CPs, with an average population of 8636 inhabitants. Temperatures oscillating between 20.9 and 31 °C and scarce rainfall concentrated between June and September characterize the weather of this city. After 1981, no dengue cases were detected for two decades. Between 2001 and 2010, only two small outbreaks occurred: 2001 (14 cases) and 2006 (136 cases). Afterwards, every year there were few cases until an outbreak of 6000 cases in 2014. From 2015 onwards, all four serotypes are present, and this area should now be considered dengue-endemic (personal communication of Toledo ME).

Data collection. In Cuba, the surveillance system from the Ministry of Health (MOH) routinely collects the following information, available at the level of municipality and CP. Epidemiological data of laboratory-confirmed dengue cases from all municipal health structures are reported in the routine surveillance system of the municipal epidemiology unit of the MOH. These are clinically suspected dengue cases that tested positive on the Ultra-micro-ELISA dengue IgM test, following the national protocol [49].

The dataset used in our analysis contains for each patient the date of onset of symptoms, the date of blood sample collection, the patient’s residential address and whether the case had alarm signs indicating a severe dengue case, based on the WHO guidelines [50]. The Cuban routine surveillance system combines a passive approach with active case finding from the moment the first arboviral disease case is confirmed. This contributes to the data quality and completeness. The data obtained cover the period of 2010–2014 for Santiago. In the case of Cienfuegos, the study period starts in 2012, coinciding with the start of regular reports of dengue cases. In both municipalities, the end of the period was marked by the start of the Zika epidemic (2015 for Santiago, 2016 for Cienfuegos).

Entomological data on *Ae. aegypti* infestation are routinely collected by the Provincial Vector Control Unit, responsible for the vector control program [51]. These data include information per month on the number of houses with at least one water-holding container with *Ae. aegypti* immature stages, number of water-holding containers with *Ae. aegypti* immature stages, number of houses visited and number of pupae detected per 100 houses for each CP. One out of three of all inspected houses is systematically revisited by a specialized provincial team as part of a quality control system implemented in the Cuban routine program to control for the motivational factor of the technicians in the area.

Socioeconomic and environmental data were not included, as these data are not available in Cuba at the level of CPs, only at the municipality level from the census conducted in 2012 [52]. Nevertheless, we consider that these factors are linked to the entomological component, which is a more direct measure of vector abundance and disease risk [18,53,54]. Demographic data are available from household census records from the National Statistics office for calculation of population density per CP and from the provincial health authorities for the sites with daytime high population densities [44].

### 2.3. Multicomponent Risk Assessment and Mapping

This section describes the methodology in three stages: (1) identifying components and indicators, (2) specifying the models and (3) identifying the methodology to classify and map the results.

#### 2.3.1. Preliminary Stage: Knowledge-Based Identification of Components and Indicators

We developed a multicomponent approach based on the Integrated Prevention and Control Strategy for Dengue in Mesoamerica (MSA), promoted by the Pan-American Health Organization (PAHO) [19]. It proposes to base dengue control strategies on the identification of geographical areas with high transmission risk using epidemiological, entomological, demographic and environmental components. An expert group of academics and policymakers from four Latin-American countries, united through the DENTARGET network (https://www.dentarget.org/, accessed on 1 March 2023), took this MSA plan as a basis for designing a proactive dengue control strategy in different Latin-American contexts. This expert group discussed which data are available in the Latin-American countries and which ones are suitable for the risk mapping proposed in the MSA plan.

To capture the variability in transmission patterns, the expert group proposed to take into account historical data from a two- to five-year window period, including as much as possible of the above-mentioned components [18]. To define the epidemiological characteristics of a spatial unit, indicators such as the cumulative incidence of cases, the typical location of the first cases detected at the beginning of the seasonal increase or outbreak, the persistence of cases in or between epidemics/seasons, the proportion of dengue serotypes circulating and the number of severe cases can be used. The entomological profile can be characterized based on persistence of high *Aedes* larval indices over time and cumulative infestation levels, and in case these are not available, environmental risk factors (such as presence of suitable places for *Aedes* infestation, e.g., tire storage areas, cemeteries, water-storage behavior of households) might be used as proxies. For what concerns the demographic components, it is suggested to complement the population density with indicators that take into account the human movement as a driver of disease dispersion [17].

Climatic and geographical factors such as temperature, rainfall, relative humidity and altitude are important to define risk, but the ability to differentiate risk levels with these variables is limited in this set-up, as they tend to vary minimally in the context of neighboring geographical areas [55,56].

Indicators for the Cuban setting are summarized in Table 1 and were the result of a discussion among researchers and members of the provincial vector control units of the Ministry of Health.

#### 2.3.2. Modeling Stage

A correlation matrix is generated to unveil possible relationships between the indicators. With the aim of assigning a level of risk of dengue transmission to each CP, we followed two approaches to include multiple components in the risk analysis: a knowledge-based one, based on the understanding or experience considering the causal relationships associated with the disease risk and leading to disease occurrence in a community [57], and a data-driven approach, relying on statistical models. In what follows, we consider the study municipality A divided into n non-overlapping CPs: ${\left\{A_{i}\right\}}_{i=1}^{n}$.

Multicomponent vulnerability index approach

This section describes the development of a multicomponent vulnerability index that encompasses the knowledge-based selection and weighing of components and indicators, hereafter called KBMCvulnerability. The expert group described above (https://www.dentarget.org/, accessed on 1 March 2023)) [18] first selected the components and decided that each component (epidemiological, entomological and demographic) is of equal importance for estimating the risk of dengue transmission. Based on their expertise they also selected the indicators included in the components. Hence, given K=3 components, each component *k* (k=1,…,K) containing $p_{k}$ indicators (Table 1), we assigned equal weights for the different components and for the indicators within the components. For each geographical entity, the CP i (i=1,…, n), we compute a vulnerability index as follows:

1. Generate *z*-score standardized indicators ($z_{ij}$) with mean 0 and standard deviation 1:
  (1) $$z_{ij}=\frac{x_{ij}-\mu_{j}}{s_{j}},$$
  where $\mu_{j}$ and $s_{j}$ are, respectively, the mean and standard deviation of indicator *j* in CP *i*.
2. Calculate an aggregated value for each CP *i* and component k (k=1,…,K) as
  (2) $$a_{ik}=\frac{1}{p_{k}}\overset{j_{p_{k}}}{\underset{j=j_{1}}{\sum }}z_{ij},$$
  where $j=j_{1},\ldots ,j_{p_{k}}$ represent the indicators included in component k.
3. Define a global index $U_{i}$ as a measure of vulnerability, by averaging the aggregated values of the *K* components:
  (3) $$U_{i}=\frac{1}{K}\overset{K}{\underset{k=1}{\sum }}a_{ik}$$
4. Rank the CPs in increasing order of $U_{i}$ and classify them using the quantile method into five classes, assigning a rank between 1 and 5, representing the level of vulnerability.

Data-driven multicomponent modeling

In addition to the above-described multicomponent vulnerability index approach (knowledge-based selection and weighing), two data-driven weighing approaches were used. The first one relies on the use of statistical regression models to relate the relative risk of disease (the outcome) with potential risk factors. Here, disease risk for each geographical unit is estimated using statistical models that incorporate standardized covariates from the area and from the neighboring areas (CPs, in our case study) to improve local estimates. The second one uses a two-stage Principal Component Analysis (PCA) to assign data-driven weights to the different components in the vulnerability index.

##### Multicomponent Multivariate Regression Modeling of Disease Incidence

In this approach, abbreviated as MCincidence, we describe the three steps of risk estimation based on multivariate regression modeling: (1) verification of model assumptions (collinearity testing), (2) selection of the model and (3) the estimation of the relative risk.

We test the presence of collinearity among predictors by computing the generalized Variance-Inflation Factors (VIFs). We excluded the indicator with the highest variance inflation factor (VIF > 10) and repeated this process to retain only the covariates with a VIF ≤ 10 (Supplementary material Text S1, Table S1).

We use Generalized Linear Mixed Models (GLMMs) for the analysis of dengue disease incidence rates and its spatial distribution. These models are useful to estimate random effects in addition to the fixed effects [58]. In disease modeling and mapping [24], the observed case counts, $y_{i}$, in area i, conditional to the relative risk $\theta_{i}$, are assumed to follow a Poisson distribution with mean $\mu_{i}=E_{i}\theta_{i}$, where $E_{i}$ is the expected case count in area i and the relative risk $\theta_{i}$ represents the Standardized Incidence Ratio (SIR) in area i, considering the entire population in the municipality as reference. This relative risk can be decomposed as:

(4) $$\log\left(\theta_{i}\right)=\beta_{0}+\beta x_{i}+\psi_{i},$$

where $\beta_{0}$ is the intercept (interpreted as the logarithm of the global risk or the mean log risk), β is the vector of *p* coefficients (fixed effects) associated with the vector of covariates $x_{i}$ in area i and $\psi_{i}$ is a random effect for area i that can take different linear forms: as an independent and normally distributed residual or as a combination of independent and spatial random effects, as in [58], the most commonly used in the literature for disease mapping [59]. The inclusion of such a random effect allows for over-dispersion in the Poisson model that would otherwise assume equal mean and variance for area i [60].

In our case studies, the indicators from Table 1 used as covariates ($x_{i}$) were as follows: proportion of severe cases, times initiating outbreak, case persistence, maximum monthly Breteau index averaged over the years of the study period, average monthly Breteau index, pupae per house index from the last epidemic year of the study period, population density and locations with high human concentration and mobility.

We expanded $\psi_{i}$ to specify structured and unstructured spatial effects, resulting in two non-spatial (GLM, Independent GLMM) and four spatial structures (Supplementary material Table S2): Intrinsic Conditional Autoregressive (ICAR), Besag, York and Mollié model (BYM), Leroux et al. model (LEROUX) and Spatial Lag Model (SLM). These models are commonly used in disease risk mapping, especially for dengue, using spatially aggregated count data [58,59,60,61,62,63,64].

The underlying spatial structure is defined through the matrix $W=\left[w_{ij}\right]$. In this paper, we assume a first-order neighborhood structure (Queen’s adjacency) and binary spatial weights, such that CPs that share a common boundary are considered neighbors. The predictors, priors and functions for each model are described in Table S2.

In the framework of Bayesian inference, the model parameters were determined using Integrated Nested Laplace Approximation (INLA), which offers a fast and accurate alternative to Markov Chain Monte Carlo (MCMC) for latent Gaussian models [65]. This approach has a wide range of applications and can be used to fit a variety of models including generalized linear mixed, spatial and spatial-temporal models [66].

For the model selection, we use the Deviance Information Criterion (DIC) [67]. It takes into account the goodness-of-fit (by means of the deviance) and incorporates the complexity of the model with a penalty term, which is defined as the effective number of parameters, similar to the Akaike Information Criterion [68,69]. The model with the smallest DIC is selected for disease risk estimation, classification and mapping.

Using the selected model, we calculate the relative risk of disease in each CP as compared to the municipality and obtain the point estimates of disease risk ($\theta_{i}$) from the INLA posterior mean, meanwhile the 95% credible intervals are obtained from the 0.025 and 0.975 quantiles of the fitted model.

##### Multicomponent Vulnerability Index

A data-driven weighting based on Principal Component Analysis (PCA) is used to obtain a multicomponent vulnerability index—abbreviated as PCAMCvulnerability. We use the three expert-identified components (epidemiological, entomological and demographic) as dimensions of the vulnerability index. As stated above, given K=3 expert-identified components and each component *k* (k=1,…,K) contains $p_{k}$ indicators (Table 1), we construct a vulnerability index using a two-stage PCA, as described elsewhere [70,71,72].

Since our goal is to construct an index, we decided to account for 100% of the total variation to avoid discarding information from the input indicators in the estimation of the overall vulnerability index. In the first stage, we use PCA to estimate three separate sub-indices: epidemiological, entomological and demographic. In the second stage, we use PCA to estimate the vulnerability index on the basis of the estimated indices of the components as input indicators.

The indicators included in each component are first standardized, as described in Equation (1), and then aggregated using PCA to derive an index for each expert-identified component (*K*). These indices are considered as the unobserved variables $I_{i}^{Ep}$, $I_{i}^{En}$ and $I_{i}^{De}$, where the superscripts denote the epidemiological, entomological and demographic components, respectively. We obtain the indices of each component for each CP *i* as weighted averages as follows:

(5) $$I_{i}^{Ep}=\frac{\sum_{j=1}^{4}\lambda_{j}^{Ep}P_{ji}^{Ep}}{\sum_{j=1}^{4}\lambda_{j}^{Ep}},$$

(6) $$I_{i}^{En}=\frac{\sum_{j=1}^{2}\lambda_{j}^{En}P_{ji}^{En}}{\sum_{j=1}^{2}\lambda_{j}^{En}},$$

(7) $$I_{i}^{De}=\frac{\sum_{j=1}^{2}\lambda_{j}^{De}P_{ji}^{De}}{\sum_{j=1}^{2}\lambda_{j}^{De}},$$

where $\lambda_{j}^{Ep}$, $\lambda_{j}^{En}$ and $\lambda_{j}^{De}$ are the eigenvalues of the *j*-th principal components and $P_{ji}^{Ep}$, $P_{ji}^{En}$ and $P_{ji}^{De}$ are the *j*-th principal component scores for CP *i*. It is assumed that $\lambda_{1}^{Ep}>\lambda_{2}^{Ep}>\ldots >\lambda_{4}^{Ep}$, $\lambda_{1}^{En}>\lambda_{2}^{En}$ and $\lambda_{1}^{De}>\lambda_{2}^{De}$. The number of terms in the summations (4, 2 and 2) represents the number of indicators included in the epidemiological, entomological and demographic components. The principal component scores $\left(P_{l}^{Ep},\;P_{l}^{En},\;P_{l}^{De}\right),\;l=1,\ldots ,p_{k},$ are estimated as linear combinations of the component loadings ($\delta_{lp}^{Ep},\;\delta_{lp}^{En},\;\delta_{lp}^{De}$) and the *p* indicators ($X_{p}^{Ep},X_{p}^{En},X_{p}^{De}$) included in the expert-identified component *K*, as follows:

(8) $$P_{l}^{Ep}=\sum \delta_{lp}^{Ep}X_{p}^{Ep},$$

(9) $$P_{l}^{En}=\sum \delta_{lp}^{En}X_{p}^{En}$$

(10) $$P_{l}^{De}=\sum \delta_{lp}^{De}X_{p}^{De}$$

The number of principal components is equal to the number of indicators included in each expert component.

In the second stage, we compute the overall vulnerability index with the following equations:

(11) $$P_{1i\;}=\varphi_{11}I_{i}^{Ep}+\varphi_{12}I_{i}^{En}+\varphi_{13}I_{i}^{De},$$

(12) $$P_{2i}=\varphi_{21}I_{i}^{Ep}+\varphi_{22}I_{i}^{En}+\varphi_{23}I_{i}^{De},$$

(13) $$P_{3i}=\varphi_{31}I_{i}^{Ep}+\varphi_{32}I_{i}^{En}+\varphi_{33}I_{i}^{De},$$

(14) $$PCIndex_{i}=\frac{\sum_{j=1}^{3}\lambda_{j}P_{ji}}{\sum_{j=1}^{3}\lambda_{j}},$$

where $PCIndex_{i}$ is the index of vulnerability for the *i*-th CP; $\lambda_{j}$: eigenvalue of the *j*-th principal component; $P_{ji}$: the *j*-th principal component for the *i*-th CP. $\varphi_{11}$, $\varphi_{12}$ and $\varphi_{13}$ are the loadings of the respective sub-indexes.

#### 2.3.3. Classification and Mapping

We classified the estimated risk and vulnerability in five classes using the quantile method, which divides the distribution of the ordinal ranking of the data into intervals of equal cardinality [73]. As a result, the same number of CPs per class is obtained, facilitating the visual comparison of the estimated parameters through the construction of thematic maps. With the results obtained, we aim to visualize the spatial risk patterns as a tool for policy use in decision making.

### 2.4. Comparison with Single-Component Mapping

Two single-component approaches commonly used in public health [74] were used: incidence and incidence relative risk mapping.

#### 2.4.1. Single-Component Cumulative Incidence Model

This model, abbreviated as SCincidence, uses a combination of disease events and population data. The spatial distribution of disease data can be displayed as cumulative incidence over the studied period at the level of the CP.

#### 2.4.2. Single-Component Standardized Incidence Ratio Model

For this model, abbreviated SCSIR, incidence risk estimates are obtained by computing the Standardized Incidence Ratio (SIR): for each area i (i=1,…,n), where ${\mathrm{SIR}}_{i}$ is defined as the ratio of the observed counts ($Y_{i}$) to the expected counts ($E_{i}$) over the study period. The expected counts $E_{i}$ represent the total number of cases expected if the population of area i would behave in the way the standard population behaves (standard population equal to population of entire municipality). $E_{i}$ is calculated using indirect standardization. ${\mathrm{SIR}}_{i}$ indicates whether area i has a higher (${\mathrm{SIR}}_{i}>1$), equal (${\mathrm{SIR}}_{i\;}=1$) or lower (${\mathrm{SIR}}_{i}<1$) risk than expected from the population at municipality level.

We compared the multicomponent knowledge-based and data-driven approaches with the single-component approaches by calculating Kendall’s tau-b rank correlation coefficient [75,76], ranging between −1 and 1, with 0 indicating no correlation between the results of the tested methods. A value of 1 indicates that the rankings assigned by the compared methods are identical, while a value of −1 indicates totally opposite rankings.

The analyses and thematic maps generated for spatial visualization were done using R (version 4.01) and RStudio (version 1.4.1717) with the packages INLA, sf, sp, spdep, ggplot2 and tmap [77,78,79,80,81].

## 3. Results

**Figure 1:**
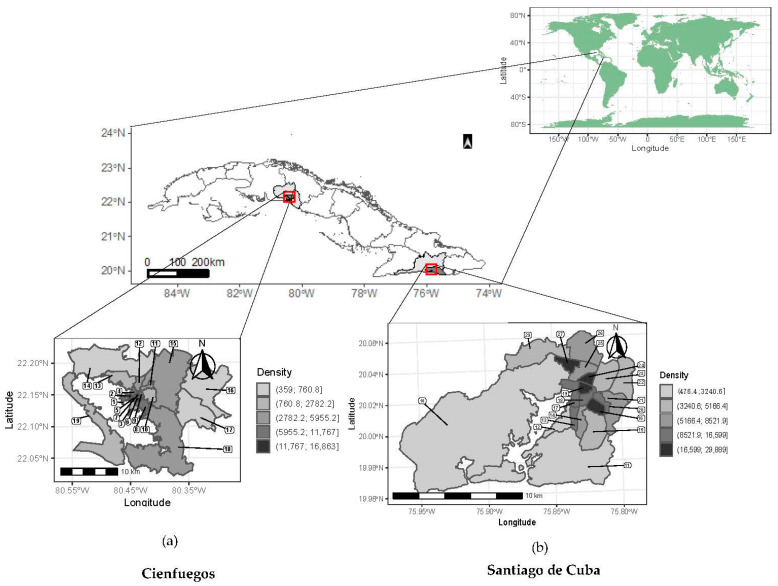
Map of CPs with their respective population densities (person per square kilometer) for Santiago de Cuba and Cienfuegos municipalities. (**a**) CPs in Cienfuegos municipality: 1 Reina, 2 Centro Histórico, 3 Punta Gorda, 4 San Lázaro, 5 La Gloria, 6 La Juanita, 7 Juanita 2, 8 Junco Sur, 9 Tulipán, 10 La Barrera, 11 Buena Vista, 12 Pueblo Griffo, 13 Pastorita, 14 Paraíso, 15 Caonao, 16 Guaos, 17 Pepito Tey, 18 Rancho Luna, 19 Castillo-CEN. (**b**) CPs in Santiago de Cuba municipality: 10 Agüero Mar Verde, 11 Ciudamar, 12 Altamira, 13 Vista Hermosa, 14 Veguita de Galo, 15 Chicharrones, 16 Flores, 17 G. Moncada, 18 J. M. Heredia, 19 Los Maceo, 20 30 de Noviembre, 21 Santa Bárbara, 22 Vista Alegre, 23 Sueño, 24 Los Olmos, 25 Mariana Grajales, 26 José Martí Norte, 27 José Martí Sur, 29 Manuel Isla.

The two studied municipalities are presented in Figure 1. High population densities are concentrated in the center of these two cities, coinciding with older neighborhoods, reaching a population density between 16,599 and 29,889 persons/km^2^ in Santiago de Cuba. In both cities, the peripheral CPs have the lowest densities, ranging between 359.0 and 760.8 persons/km^2^ in Cienfuegos.

**Figure 2:**
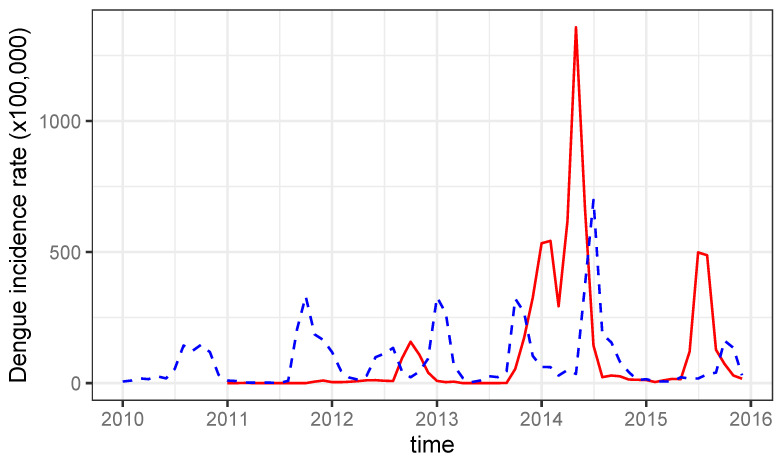
Monthly incidence rates of dengue (per 100,000) in Santiago de Cuba (dashed blue) and Cienfuegos (solid red) municipality over the period of 2010–2015.

Between 2010 and 2015, dengue incidence shows a clear seasonal pattern in both municipalities. In particular, in Santiago de Cuba, each year there is a wave starting in summer and lasting for 6–7 months, while in Cienfuegos, waves are smaller and less frequent in the first years. In both municipalities, the increasing trend in the period included a large outbreak in 2014 (Figure 2).

The CP level indicators from the two study areas were tested for multicollinearity by computing the VIFs. Average monthly BI over five years was the indicator with the highest VIF (VIF = 32.126) in Santiago de Cuba and the second highest in Cienfuegos (VIF = 15.082). It was thus not used in the further analysis. After removing this indicator, no other variable showed a VIF greater than 10. Table S1 shows a summary of the VIFs of the variables from the two study sites.

**Figure 3:**
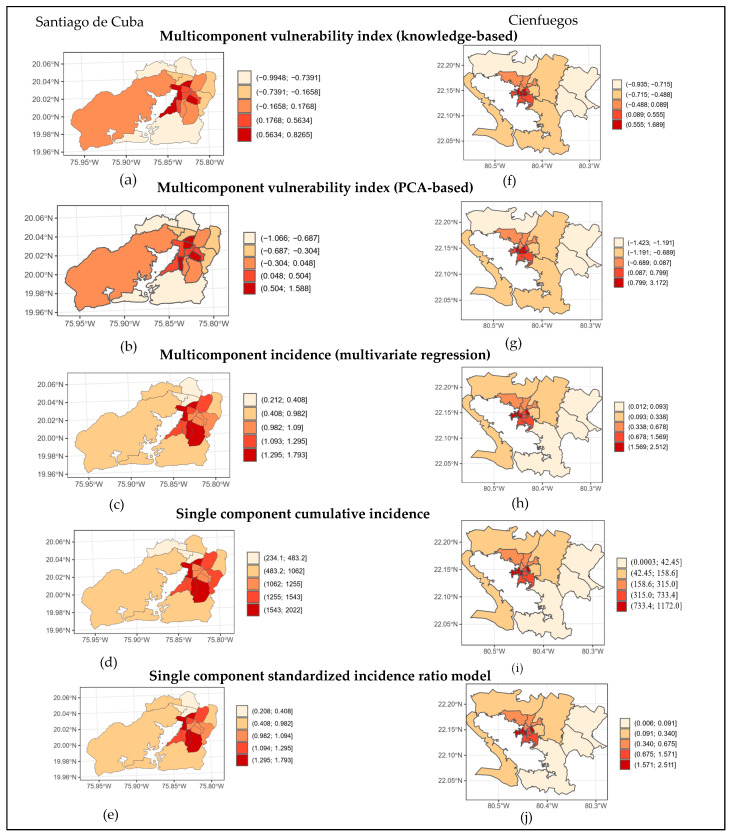
Dengue risk maps based on KBMCvulnerability, PCAMCvulnerability, MCincidence, SCincidence and SCSIR models in Santiago de Cuba and Cienfuegos. (**a**) KBMCvulnerability index in Santiago de Cuba; (**b**) PCAMCvulnerability index in Santiago de Cuba; (**c**) MCincidence in Santiago de Cuba; (**d**) SCincidence in Santiago de Cuba; (**e**) SCSIR in Santiago de Cuba; (**f**) KBMCvulnerability index in Cienfuegos; (**g**) PCAMCvulnerability index in Cienfuegos; (**h**) MCincidence in Cienfuegos; (**i**) SCincidence in Cienfuegos; (**j**) SCSIR in Cienfuegos.

In Santiago de Cuba, the KBMCvulnerability model identifies Los Olmos, Guillermón Moncada, Altamira and Flores as the CPs with the highest vulnerability to dengue (Table 2 and Figure 3). In these CPs, the score of the epidemiological component ranked between three and five and the entomological component received the maximum value in three out of four CPs. In Cienfuegos, the CPs with the highest ranks were La Juanita, La Gloria, Centro Histórico and Juanita 2. In these areas, the three components show almost equal importance: three out of four CPs received a rank of five in the epidemiological and entomological component, while the demographic dimension always received the maximum rank.

As a first step in the data-driven MCincidence approach, the collinearity between the predictors was evaluated. This resulted in the exclusion of one entomological indicator, the average monthly Breteau index. With the generalized linear mixed models, following a Bayesian framework and INLA, several models (FIXED, IID, ICAR, BYM, BYM2, LEROUX, SLM) were fit with different specifications of the random terms and the spatial structure (Supplemental Material Text S1, Table S1).

Table 3 shows that the values of Deviance Information Criterion (DIC), the effective number of parameters (p.eff) and the Watanabe–Akaike Information Criterion (WAIC) are very similar for the models including random effects and lower than the values of the model with only fixed effects. This suggests that including random effects in the models is necessary to explain the variability of the data. Based on the DIC and WAIC, BYM and ICAR were the best models for Santiago de Cuba and Cienfuegos, respectively. With these two models, the Relative Risk (RR) of disease transmission was estimated taking into account the effect of epidemiological, entomological and demographic indicators, and a structured spatial random effect.

The results of fitting the selected models to the data of Santiago de Cuba and Cienfuegos using the MCincidence approach are shown in Table 4. The analysis of the coefficients of the indicators included in the models suggests that persistency of cases is the only indicator that is statistically significantly associated with a positive increase in disease risk, meaning that an increase of one unit in the standardized persistence is associated with an increase of 26% and 36% in the risk of dengue disease in Cienfuegos and Santiago de Cuba, respectively, holding the rest of the covariates constant.

The ranks created for the spatial units under study in both municipalities using the vulnerability (KBMCvulnerability and PCAMCvulnerability) and the risk (MCincidence, SCincidence and SCSIR) models are shown in Table 5 and Figure 3. These show that despite the different methodologies underlying them, all models identified Los Olmos in Santiago de Cuba, and La Gloria and Centro Histórico in Cienfuegos, as the most vulnerable areas.

There is a high correlation between the vulnerability risk stratification obtained by the knowledge-based multicomponent and data-driven principal component analysis approaches, with a Kendall’s tau of 0.89 and 1 for Santiago de Cuba and Cienfuegos, respectively (Table 6). The correlation between the incidence-based approaches—MCincidence, SCincidence and SCSIR—had tau values between 0.9 and 1 for Santiago de Cuba. For Cienfuegos, there was a perfect agreement between the three incidence-based models. The agreement between the vulnerability risk stratification and the MCincidence-based models was below 0.6 for Santiago de Cuba and reached 0.84 for Cienfuegos.

### Risk Mapping

Five maps were created with the models proposed in each city. The dengue risk maps show that CPs with higher vulnerability/risks are concentrated in the center of the respective cities (Figure 3).

## 4. Discussion

Two single-component approaches (cumulative incidence and standardized incidence ratio models) were compared with (1) a multicomponent model predicting dengue incidence and incorporating epidemiological, entomological and demographic indicators; and (2) two multicomponent approaches where a vulnerability index was estimated by giving different weights to the epidemiological, entomological and demographic components. The first approach was based on expert information to select and weigh the indicators used to obtain quantitative vulnerability estimates [57], and the second was data-driven based on PCA. The data-driven multivariate regression approach used generalized linear mixed models with random effects (BYM and ICAR) in a Bayesian framework fitted with INLA. For all analyses, routine data from two Cuban municipalities over the period of 2010–2015 were used and the spatial heterogeneity pattern of the dengue transmission was visualized using thematic maps. The result of the risk mapping was discussed with the provincial dengue control teams, who confirmed the presence in these areas of multiple environmental risks and human risky behaviors concerning hygiene and water management.

The comparison of the ranks of disease risk obtained by the three models using mainly—and almost only—incidence data (SCincidence, SCSIR and MCincidence) showed similar results, while the ranks obtained by the multicomponent vulnerability approaches (KBMCvulnerability and PCAMCvulnerability), giving more attention to entomological and demographic information, besides incidence data, were somehow different. The vulnerability approach differed more from the incidence-based approaches in Santiago de Cuba than in Cienfuegos, which could be explained by a longer history of dengue circulation and immunity influencing the clinical presentation and transmission, as well as more relative importance of entomology and demography in explaining transmission in the former municipality.

Disease risk mapping can have different goals, ranging from the visualization of the extent of a disease to providing support for the implementation of public health interventions, and can use, from descriptive or analytical maps based on logistic regression, generalized linear models to machine learning methods (e.g., based on Random Forest) [25,82,83]. Advances in geospatial science have supported public health programs in their planning of disease control and elimination activities with the identification of disease clusters or “hotspots” and also with the production of spatial prediction maps [15]. These maps can be used for targeting vector control or other interventions [83] because it has been observed that massive campaigns of mosquito control with full coverage of the cities are not sustainable in practice, especially in resource-constrained countries [42]. Evidence on the use of disease risk mapping to identify areas of higher vulnerability for dengue transmission with the aim of guiding preventive actions is less extensive and still shows research gaps [83]. In Colombia, vulnerable areas were identified using socio-demographic data [30], while in Argentina and Brazil, multicomponent models have been automated and implemented using software tools [84,85].

The multicomponent methods used in this study have the advantage that they capture information from multiple dimensions of the complex network of factors that are related to dengue transmission. The knowledge-based approach is fairly simple and easy to implement, as it does not require statistical tools. Nevertheless, it requires more data than the incidence-only method, and uncertainty can only be assessed by bootstrapping [86,87].

For the data-driven modeling approach, generalized linear mixed models can be used with different distributions of the data, including those in the exponential family (binary, binomial, Poisson, normal, gamma). A Bayesian framework can be used to fit them while taking into account different aspects of the relations between the factors associated with the research problem and the hierarchical structure of this relation.

There are several ways to specify and fit the models using a Bayesian framework. We fitted the models using INLA, a fast and computationally efficient alternative to MCMC. In spatial analysis, this technique can use indicators of autocorrelation to strengthen the models by borrowing information from the neighboring areas and is especially useful when data availability is limited. Its drawback is that more advanced statistical knowledge and computational skills are needed. For the selection of the best model, we used DIC. DIC has been recommended, as it is based on the model fit and the complexity of the model [88]. An alternative data-driven approach is to assign weights using PCA. It has been employed to create an index that takes into account the multi-dimensionality of the problem and uses weights extracted from the datasets [86].

In contrast to the vulnerability multicomponent approaches, disease occurrence maps are often used since incidence is often the only data available. These maps are used to identify areas that concentrate a large fraction of *Aedes*-borne disease cases and are regarded as a proxy for transmission risk. The disadvantages of this approach are that it does not represent asymptomatic cases and misses cryptic/silent transmission and since place of residence is used for case reporting, it might overlook the actual place of transmission. Moreover, as incidence data depend on the health-seeking behavior of people, in underserved areas with less health care attention, this may have a negative impact on the reliability of spatial heterogeneity risk maps [89].

Epidemiological and entomological data that are collected routinely have been used by several authors to design spatial decision support systems for the early detection of outbreaks [90,91]. Nevertheless, adequate quality and extent of such geo-referenced data, crucial elements for developing reliable risk maps, are still lacking in many health systems, in addition to the difficulties in the analysis and use of these maps [25]. The scale is another key factor. It has been suggested that modeling of environmental land-use data at country scales is important for guiding national programs but is not useful at decentralized levels, where control actions are planned and implemented [92].

However, a strength of this study is the use of routinely collected surveillance data. The existence of fine-grained georeferenced monthly repeated measurements for several years offers a unique opportunity to analyze epidemiological and entomological information. Such data can be subject to bias, but the Cuban surveillance system puts in place actions to guarantee quality and completeness. Underreporting of symptomatic cases is unlikely to be substantial in the Cuban context, as the routine Cuban surveillance system combines a passive approach with active case finding from the moment the first dengue or other arboviral disease case is confirmed [47]. A limitation, though, is that confirmed dengue cases are registered according to the residential addresses, which is not necessarily the place of infection, especially because *Ae. aegypti* has a biting preference during the day, when many people are outside their homes.

Likewise, the entomological information is routinely collected by vector control technicians during their monthly inspection of all houses using standardized procedures, and completeness of information depends on their motivation. Additionally, a quality control system is implemented in the Cuban routine program to control for this factor and consists of a systematic revisiting of one-third of all inspected houses by a specialized provincial team.

Although there is growing evidence suggesting that human mobility plays an important role in the transmission of dengue [93,94,95], it was not taken into account, as it is not routinely collected and therefore not readily available. Another limitation is that we did not include meteorological data, though weather has a major influence on the temporal pattern of an epidemic [56].

The strategies for modeling and mapping of transmission risks for dengue are under debate, as there are no dengue-specific thresholds defined and widely accepted, neither for entomological indicators or incidence figures [50,96,97,98,99], which makes multicomponent risk mapping important. Our analyses and the evidence of hotspot presence and persistence from other studies provide a logical framework for guiding the prioritization of preventive control actions [26]. Hotspot detection must be a flexible and interpretable approach, as risks, pathogens and human behavior can change. The analysis of the spatio-temporal patterns underlying the spread of dengue and hotspot detection may provide useful information to support public health officers to control and predict dengue spread over critical hotspots. Such hotspots can be targeted with preventive and long-lasting interventions before the peak transmission period.

No single approach is likely to be optimal for every question, and models need to take into account the local context and data availability. The small difference between single- and multicomponent incidence maps indicates that in a setting with a narrow availability of data, simpler models can be used. Nevertheless, the incidence-based multicomponent model (which implements a generalized linear mixed model) allows for a robust parametrization with factors associated with disease endemicity and provides more information by means of the covariate-adjusted and spatially smoothed RR of disease transmission, which can be used for the prospective evaluation of interventions. In addition, several shortcomings of the simpler approaches have been reported. For instance, extremely high values of Standardized Mortality Ratios (SMRs)—a measure similar to SIR—were found by Wakefield, probably caused by small expected numbers in large populated areas [100]. Additionally, he identified difficulties with the associated *p*-values since statistically significant results were also obtained even with small deviations from 1 in largely populated areas, among other drawbacks. However, if a vulnerability model is used, where different origins of risk (demographical, number of cases, mosquito density) are important, the resulting risk maps might be quite different from the incidence-based ones. The vulnerability approach that we followed includes an indicator of human mobility and potential for out-of-residence transmission, which could explain part of the disagreement with the risk (incidence) approach.

The choice of maps—and indicators included—must be guided by objectives [101]: does one need a map for visualization of disease distribution and intensity, for implementing preventive or reactive vector control measures or for prediction of concentration of cases for better organization of health care? A prospective validation of the used approaches has been suggested, although this is still an issue of debate [32,35,36,40]. For the incidence-based models, one can use the incidence of the subsequent years to evaluate agreement of future and past incidences. However, for the vulnerability maps, one cannot simply compare subsequent maps of disease incidence, as vulnerability or transmission risk is not captured by a single indicator but needs to include the multi-factorial complexity. Nevertheless, an approach for the validation of the indices, with limitations though, is to use disease incidence as a proxy of the true vulnerability [40,102]. Linear regression and Receiver Operating Characteristic (ROC) curves have also been used for the statistical validation of the indices [102,103,104]. Additionally, dengue hotspots identified using vulnerability indices have been validated by comparing their distribution with the distribution of Zika and chikungunya [26,48]. Still, validation of a vulnerability map should preferably be done with an intervention trial targeting prevention and control measures to the high-risk areas by observing whether transmission is decreasing or not.

For a hotspot-driven approach at the sub-city level to be viable and effective, it is critical to determine whether the observed heterogeneity at this scale is a feature of dengue transmission or whether it follows a more stochastic pattern [105]. Therefore, to achieve robust policy recommendation, prospective validation is not only needed for the evaluation of the effectiveness of the interventions but also to detect potential changes in the distribution of hotspots due to the targeted interventions or the changes in demographic or epidemiological trends, which would have to be addressed in an adaptive and iterative process.

In conclusion, caution is needed when interpreting maps, as the results vary depending on the importance given to the components involved in disease transmission. The multicomponent vulnerability mapping needs to be prospectively validated based on an intervention trial targeting high-risk areas.

## Figures and Tables

**Table 1:** CP-level transmission-linked indicators included in the multicomponent models.

Component	Indicators	Description	Source *
Epidemiological	Cumulative number of dengue cases	Cumulative number of confirmed dengue cases	Based on retrospective surveillance data from 2010–2015 on dengue cases
	Cumulative incidence of dengue cases	Cumulative number of confirmed dengue cases per 10,000 inhabitants	
	Proportion of severe dengue cases	Proportion of severe cases among confirmed dengue cases	
	Times initiating outbreak	Number of times that dengue seasonal increase started in CP	
	Dengue case persistence	Number of months with more than five cases per month	
Entomological	Maximum monthly Breteau index averaged over the years of the study period	Breteau index of the month with highest *Aedes* infestation per year, averaged over time ^1^	Based on retrospective entomological surveillance data from 2010–2015
	Average monthly Breteau index	Average monthly Breteau index over time	
	Pupae per house index	Pupae per house index from the last epidemic year of the study period (2014) ^2^	
Demographic	Population density (per square km)	Population divided by the surface in square kilometers	National Statistics Office, Provincial Office of the Ministry of Health
	Locations with high human concentration and mobility	Number of locations within a CP with intense daytime human mobility and concentration. These locations were identified by local knowledgeable field workers based on their qualitative appraisal of heavy circulation or prolonged presence of persons and selected by consensus. They included, among others, schools, factories, health centers, transportation nodes and markets.	

^1^ Breteau index calculated as the number of water-holding containers with *Ae. aegypti* immature stages per 100 houses. ^2^ Pupae per house index calculated as the total number of pupae found divided by the total number of inspected households. * The indices and models for Santiago de Cuba cover five years (2010–2014), and those for Cienfuegos cover four years (2012–2015).

**Table 2:** Multicomponent score ranks, estimated by the KBMCvulnerability index, per CP in Santiago de Cuba and Cienfuegos.

CP	Epidemiological Z (R) ^1^	Entomological Z (R) ^1^	Demographic Z (R) ^1^	KBMCvulnerability Index ^2^, Z (R) ^1^
Santiago de Cuba				
Los Olmos	0.34 (4)	1.02 (5)	1.12 (5)	0.83 (5)
Guillermón Moncada	1.17 (5)	0.04 (3)	1.02 (5)	0.74 (5)
Altamira	1.13 (5)	1.32 (5)	−0.39 (2)	0.69 (5)
Flores	0.23 (4)	1.19 (5)	0.42 (4)	0.61 (5)
Vista Hermosa	0.14 (3)	1.75 (5)	−0.24 (3)	0.55 (4)
J. M. Heredia	−0.12 (3)	−0.36 (2)	1.75 (5)	0.42 (4)
Los Maceos	0.41 (4)	−0.21 (3)	0.65 (5)	0.28 (4)
30 de Noviembre	−0.06 (3)	0.43 (4)	0.19 (4)	0.19 (4)
Chicharrones	0.76 (5)	0.35 (4)	−0.6 (2)	0.17 (3)
Veguita de Galo	0.42 (5)	0.56 (4)	−0.78 (1)	0.07 (3)
Aguero Mar Verde	0.26 (4)	0.17 (4)	−0.54 (2)	−0.04 (3)
Sueño	−0.15 (2)	−0.14 (3)	−0.03 (3)	−0.1 (3)
Mariana Grajales	−0.5 (2)	−0.1 (3)	−0.18 (3)	−0.26 (2)
Santa Bárbara	−0.09 (3)	−0.97 (1)	0.03 (4)	−0.34 (2)
Vista Alegre	−0.62 (2)	−0.48 (2)	−0.06 (3)	−0.39 (2)
José Martí Sur	−0.6 (2)	−1.57 (1)	−0.02 (4)	−0.73 (2)
Manuel Isla	−0.78 (1)	−0.87 (2)	−0.67 (1)	−0.78 (1)
José Martí Norte	−0.83 (1)	−1.38 (1)	−0.54 (2)	−0.92 (1)
Ciudamar	−1.12 (1)	−0.75 (2)	−1.12 (1)	−0.99 (1)
Cienfuegos				
La Juanita	1.34 (5)	2.28 (5)	1.45 (5)	1.69 (5)
La Gloria	1.06 (5)	2.12 (5)	0.97 (5)	1.38 (5)
Centro Histórico	0.93 (5)	0.2 (4)	2.3 (5)	1.15 (5)
Juanita 2	0.44 (4)	0.69 (5)	0.75 (5)	0.63 (5)
Reina	0.74 (5)	0.59 (4)	0.29 (4)	0.54 (4)
Punta Gorda	0.3 (4)	0.71 (5)	0.03 (3)	0.35 (4)
Junco Sur	0.16 (3)	0.29 (4)	0.1 (4)	0.18 (4)
Tulipán	0.49 (4)	−0.29 (3)	0.23 (4)	0.15 (4)
San Lázaro	0.19 (4)	0.24 (4)	−0.27 (3)	0.05 (3)
Buena Vista	0.16 (3)	−0.14 (3)	−0.57 (2)	−0.19 (3)
Pastorita	−0.22 (3)	−0.56 (3)	−0.36 (3)	−0.38 (3)
Pueblo Griffo	−0.44 (2)	−0.2 (3)	−0.74 (2)	−0.46 (3)
Caonao	−0.58 (2)	−0.93 (1)	−0.06 (3)	−0.53 (2)
La Barrera	−0.12 (3)	−0.63 (2)	−0.85 (2)	−0.53 (2)
Rancho Luna	−0.93 (1)	−0.97 (1)	0.08 (4)	−0.61 (2)
Castillo-CEN	−0.89 (2)	−0.66 (2)	−0.48 (2)	−0.67 (2)
Paraíso	−0.76 (2)	−0.92 (2)	−0.95 (1)	−0.87 (1)
Guaos	−0.93 (1)	−0.92 (1)	−0.95 (1)	−0.93 (1)
Pepito Tey	−0.93 (1)	−0.91 (2)	−0.96 (1)	−0.94 (1)

^1^ Z: z-score value of the standardized scores, R: rank value. The CPs in the table are ordered by the z-score of the multicomponent index. ^2^ Index: KBMCvulnerability standardized index.

**Table 3:** Comparison of the MCincidence multivariate regression models based on the Deviance Information Criterion (DIC), effective number of parameters (p.eff) and Watanabe–Akaike Information Criterion (WAIC) in Santiago de Cuba and Cienfuegos.

	Cienfuegos			Santiago de Cuba		
Model	DIC	p.eff	WAIC	DIC	p.eff	WAIC
FIXED	1112.03	−112.87	2056.54	1399.36	−129.65	2385.63
IID	164.66	18.05	160.38	202.74	18.88	197.03
ICAR	163.38	17.86	158.29	202.82	18.88	197.22
BYM	164.67	18.08	160.35	202.74	18.88	197.03
BYM2	163.85	17.90	159.08	202.76	18.89	197.05
LEROUX	163.80	17.99	158.89	202.77	18.88	197.08
SLM	163.96	18.07	159.05	202.76	18.89	197.07

**Table 4:** Posterior means, standard deviation and 95% credibility intervals for the fixed effects of the intercept and the covariates included in the MCincidence multivariate regression models.

	Cienfuegos ^2^		Santiago de Cuba ^3^	
Coefficients ^1^	Mean ^4^ (SD)	(LL; UL) ^4^	Mean (SD)	(LL; UL)
(Intercept)	0.36 (0.077)	(0.304; 0.412)	0.87(0.079)	(0.74; 1.02)
Population density	1.01 (0.483)	(0.372; 2.56)	1.03 (0.095)	(0.85; 1.25)
Locations with high human concentration	1.22 (0.233)	(0.77; 1.954)	1.07 (0.087)	(0.90; 1.27)
Maximum monthly Breteau index	0.72 (0.549)	(0.234; 2.108)	1.17 (0.125)	(0.91; 1.50)
Pupae per house index	1.83 (0.485)	(0.695; 4.843)	1.05 (0.112)	(0.84; 1.31)
Proportion of severe cases	0.94 (0.249)	(0.572; 1.552)	0.87 (0.108)	(0.70; 1.07)
Times initiating outbreak	0.78 (0.483)	(0.302; 2.085)	0.96 (0.099)	(0.79; 1.17)
Dengue case persistence	3.16 (0.6)	(1.022; 11.132)	1.36 (0.145)	(1.02; 1.82)

^1^ Standardized indicators, ^2^ ICAR model, ^3^ BYM model, ^4^ exponentiated coefficients, SD: standard deviation; LL: lower limit of the credibility interval (0.025 quantile); UL: upper limit of the credibility interval (0.975 quantile).

**Table 5:** Risk and vulnerability ranks estimated by KBMCvulnerability, PCAMCvulnerability, MCincidence, SCincidence and SCSIR models.

	Vulnerability Models		Risk Models		
CPs	KBMCvulnerability Index	PCAMCvulnerability Index	MCincidence	SCincidence	SCSIR
*Santiago de Cuba*					
Guillermón Moncada	5	4	5	5	5
Veguita de Galo	3	3	5	5	5
Los Olmos	5	5	5	5	5
Chicharrones	3	3	5	5	5
Sueño	3	3	4	4	4
Altamira	5	4	4	4	4
Vista Hermosa	4	5	4	4	4
Los Maceo	4	5	4	3	4
J. M. Heredia	4	4	3	3	3
Flores	5	5	3	3	3
30 de Noviembre	4	4	3	3	3
Santa Barbara	2	2	3	4	3
Vista Alegre	2	2	2	2	2
Aguero Mar Verde	3	3	2	2	2
Ciudamar	1	1	2	2	2
Manuel Isla	1	1	2	1	2
José Martí Sur	2	2	1	1	1
José Martí Norte	1	1	1	2	1
Mariana Grajales	2	2	1	1	1
*Cienfuegos*					
La Gloria	5	5	5	5	5
Reina	4	4	5	5	5
Centro Histórico	5	5	5	5	5
Tulipán	4	4	5	5	5
La Juanita	5	5	4	4	4
Juanita 2	5	5	4	4	4
Punta Gorda	4	4	4	4	4
Junco Sur	4	4	4	4	4
San Lázaro	3	3	3	3	3
Pueblo Griffo	3	3	3	3	3
Buena Vista	3	3	3	3	3
Pastorita	3	3	3	3	3
Caonao	2	2	2	2	2
Paraíso	1	1	2	2	2
La Barrera	2	2	2	2	2
Castillo-CEN	2	2	2	2	2
Rancho Luna	2	2	1	1	1
Pepito Tey	1	1	1	1	1
Guaos	1	1	1	1	1

**Table 6:** Kendall’s tau-b correlation for the agreement of the risk estimates between KBMCvulnerability, PCAMCvulnerability, MCincidence, SCincidence and SCSIR approaches.

	Vulnerability Models		Risk Models		
	KBMCvulnerability Index	PCAMCvulnerability Index	MCincidence	SCincidence	SCSIR
*Santiago de Cuba*					
KBMCvulnerability index	1 ***	0.89 ***	0.55 *	0.5 *	0.55 *
PCAMCvulnerability index	0.89 ***	1 ***	0.51 *	0.43	0.51 *
MCincidence	0.55 *	0.51 *	1 ***	0.9 ***	1 ***
SCincidence	0.5 *	0.43	0.9 ***	1 ***	0.9 ***
SCSIR	0.55 *	0.51 *	1 ***	0.9 ***	1 ***
*Cienfuegos*					
KBMCvulnerability index	1 ***	1 ***	0.84 ***	0.84 ***	0.84 ***
PCAMCvulnerability index	1 ***	1 ***	0.84 ***	0.84 ***	0.84 ***
MCincidence	0.84 ***	0.84 ***	1 ***	1 ***	1 ***
SCincidence	0.84 ***	0.84 ***	1 ***	1 ***	1 ***
SCSIR	0.84 ***	0.84 ***	1 ***	1 ***	1 ***

*: 0.01 < *p*-value ≤ 0.05, ***: *p*-value ≤ 0.001.

## Data Availability

Restrictions apply to the availability of the data used in this research. We received permission to use the data, but they remain in the ownership of the Ministry of Health. Data could be made available upon reasonable request to the first author with the permission of the MOH.

## Supplementary Materials

The following supporting information can be downloaded at: https://www.mdpi.com/article/10.3390/tropicalmed8040230/s1, Text S1. Checking for multicollinearity. Table S1. Variance inflation factor of indicators included in the models as covariates. Table S2. Structure and description of the models implemented in this study for dengue risk mapping.
