# Supplementary material for: The Potential of Surveillance Data for Dengue Risk Mapping: An Evaluation of Different Approaches in Cuba
Source: Trop Med Infect Dis. 2023 Apr 18;8(4):230. doi: 10.3390/tropicalmed8040230 (PMC10143650; doi:10.3390/tropicalmed8040230)
Supplement: Supplementary file 1 [file tropicalmed-08-00230-s001.zip › tropicalmed-2248066-supplementary.pdf]

Text S1. Checking for Multicollinearity**.**

To test for the presence of collinearity among predictors we first fitted a linear model of the form:

$$y_i = \beta_0 + \beta X_i,$$

where y_i is the cumulative number of cases and the vector of covariates X_i includes the following indicators from Table 1: proportion of severe cases, times initiating outbreak, case persistence cumulative, maximum monthly Breteau index averaged over the years of the study period, average monthly Breteau index, pupae per house index from the last epidemic year of the study period, population density and locations with high human concentration and mobility.

After fitting this model, we then computed the generalized variance-inflation factors (VIF) for each indicator. The indicator with the highest variance inflation above 10 (VIF > 10) was removed and then we repeated this process to retain only the covariates with VIF <= 10 (Table S1).

Average monthly BI over five years was the indicator with the highest VIF (VIF=32.126) in Santiago de Cuba, and the second highest in Cienfuegos (VIF=15.082). After removing this covariate, collinearity was resolved in both datasets.

Table S1. Variance inflation factor of indicators included in the models as covariates.

	**Cienfuegos**		**Santiago de Cuba**	
**Variables**	**VIF Before**	**VIF After excluding (AMBI5*)**	**VIF Before**	**VIF After excluding (AMBI5*)**
Population density	5.699746	5.491146	1.72912	1.365011
Locations with high human concentration	1.685777	1.559970	1.725831	1.132089
Maximum monthly Breteau index (BI)	8.062243	7.118032	20.398597	2.342819
Average monthly BI over five years	14.540425	--	32.125954	--
Pupae per House Index	15.082059	7.190873	5.194644	1.836846
Proportion of severe cases	1.946183	1.569980	1.775636	1.715571
Times initiating outbreak	5.378435	5.178661	1.993382	1.481455
Dengue case persistence	6.179897	6.048083	3.17202	3.152469

***AMBI5:** Average monthly BI over five years

Table S2. Structure and description of the models implemented in this study for dengue risk mapping.

Model	Type	Parameter inference method	Abbreviations	Parameters[g]
1.Generalized linear model[a] (Agresti, Allan, 2013)	Non spatial	Bayesian Maximum Likelihood	FIXED.EFF (GLM)	β
2.Independent, GLMM[b] (Besag, Julian et al.,1991)	Non spatial	Bayesian	IID	β, v_i
3.Intrinsic Conditional Autoregressive[c] (Besag, Julian et al.,1991)	Spatial	Bayesian	ICAR	β, u_i
4.Besag, York and Mollié model[d] (Besag, Julian et al.,1991)	Spatial	Bayesian	BYM	β, v_i, u_i
5. Leroux[e] (Leroux, Brian G et al.,2000)	Spatial	Bayesian	LEROUX	β, u_i, ρ
6. Spatial Lag Model[f] (Cliff and Ord, 1973; Ord, 1975; Bivand, 1984; Anselin, 1988; LeSage and Pace, 2009; Goméz-Rubio et al.,2015.)	Spatial	Bayesian	SLM	β, v_i, ρ

[a] Poisson regression: $y_i|\theta_i \sim \text{Poisson}(\mu_i = E_i\theta_i), \log(\mu_i) = \log(E_i) + \beta_0 + \beta X_i$

[b] Poisson Mixed Effect Independent Model: $y_i|\theta_i \sim \text{Poisson}(\mu_i = E_i\theta_i), \log(\mu_i) = \log(E_i) + \beta_0 + \beta X_i + v_i, v_i \sim N(0, \tau_v^2)$

[c] Poisson Intrinsic Conditional Autoregressive spatial model: $y_i|\theta_i \sim \text{Poisson}(\mu_i = E_i\theta_i), \log(\mu_i) = \log(E_i) + \beta_0 + \beta X_i + Zu_i + e,\ \ u \sim N(0, \sigma_u^2\Sigma),\ \Sigma^{-1} = \text{diag}(N_i) - W.$

[d] Poisson Besag, York and Mollié Spatial Model: $y_i|\theta_i \sim \text{Poisson}(\mu_i = E_i\theta_i), \log(\mu_i) = \log(E_i) + \beta_0 + \beta X_i + u_i + v_i\ , v_i \sim N(0, \tau_v^2), u \sim N(0, \sigma_u^2\Sigma), \Sigma^{-1} = \text{diag}(N_i) - W.$

[e] Leroux Spatial Model: $y_i|\theta_i \sim \text{Poisson}(\mu_i = E_i\theta_i), \log(\mu_i) = \log(E_i) + \beta_0 + \beta X_i + Zu_i, u \sim N(0, \sigma_u^2\Sigma),\ \Sigma^{-1} = \frac{1}{\tau}\left(I_n - \frac{\rho}{\lambda_{max}}C\right) = \frac{1}{\tau}\left((1-\rho)I_n + \rho M\right).$

[f] Spatial Lag Model: $y_i|\theta_i \sim \text{Poisson}(\mu_i = E_i\theta_i), \log(\mu_i) = (I - \rho W)^{-1}(X\beta + e) + \log(E_i)\ , e \sim N(0, \sigma^2 I)$

[g] β= regression coefficients for fixed effect; v_i = region-specific random effect; u_i = spatial random effect; parameter $\rho \in (0,1)$ specifies the degree of spatial dependency. Other variables used in the models: y_i = observed dengue cases number in the i-th area; X_i the vector of predictors for area i; μ_i = mean parameter of the Poisson distribution; E_i = expected count of disease in the ith area (offset); θ_i represents disease risk in spatial unit i, also known as the standardized incidence ratio (SIR) in CP i. W is the spatial weight matrix. The indicators used as predictors (X_i) were: proportion of severe cases, times initiating outbreak, case persistence, maximum monthly Breteau index averaged over the years of the study period, average monthly Breteau index, pupae per house index from the last epidemic year of the study period, population density and locations with high human concentration and mobility.
